# Deciphering organotropism reveals therapeutic targets in metastasis

**DOI:** 10.3389/fcell.2025.1677481

**Published:** 2025-10-10

**Authors:** Jhommara Bautista, Andrés López-Cortés

**Affiliations:** Cancer Research Group (CRG), Faculty of Medicine, Universidad de Las Américas, Quito, Ecuador

**Keywords:** metastasis, organotropism, pre-metastatic niche, phenotypic plasticity, metabolic reprogramming, immune evasion

## Abstract

Metastasis remains the principal cause of cancer-related mortality, yet its distribution across organs is far from random. Instead, tumor cells exhibit *organotropism*, a consistent preference for colonizing specific distant tissues, a phenomenon shaped by anatomical constraints, molecular crosstalk, and microenvironmental compatibility. Far beyond mere mechanical entrapment in vascular beds, metastatic dissemination reflects a coordinated interplay between tumor-intrinsic programs and organ-specific niches. Tumor-derived extracellular vesicles, cytokines, and matrix-remodeling enzymes actively precondition distant sites through pre-metastatic niche formation, creating permissive microenvironments primed for colonization. Simultaneously, tissue-specific immune landscapes, stromal compositions, and mechanical architectures determine the fate of disseminated tumor cells, whether they are eliminated, enter dormancy, or form macrometastases. Phenotypic plasticity, metabolic reprogramming, and immune evasion mechanisms equip subclones with the capacity to exploit these unique niches. Across cancer types, reproducible patterns of organotropic metastasis not only guide clinical surveillance and therapeutic stratification but also reveal vulnerabilities in the metastatic cascade. This review synthesizes emerging mechanistic insights across anatomical, immunological, and molecular domains to construct a comprehensive framework of organotropism, highlighting therapeutic opportunities to intercept metastasis at organ-specific checkpoints.

## Introduction

Metastasis, the principal cause of cancer-related mortality, is not a stochastic process but rather a highly organized, multistep cascade culminating in the colonization of specific distant organs (López-Cortés et al., 2022). This phenomenon, termed organotropism, reflects a non-random and reproducible pattern of metastatic dissemination that varies across tumor types and subtypes. Breast cancer metastasizing to bone, prostate cancer to the axial skeleton, and pancreatic cancer to the liver exemplify this preferential organ colonization, a concept first articulated by Stephen Paget’s “seed and soil” hypothesis in 1889 and further refined by subsequent studies emphasizing both tumor-intrinsic properties and the receptive nature of target tissues (Gao et al., 2019; Carrolo et al., 2024; Dai et al., 2025).

The anatomical layout of the vasculature offers a partial explanation for organotropism. Circulatory patterns govern initial dissemination routes, where organs such as the liver and lungs frequently receive the first wave of circulating tumor cells (CTCs) due to portal or systemic flow (Carrolo et al., 2024; Fares et al., 2020; Dai et al., 2025). Yet, hemodynamic considerations alone fail to account for the diversity of metastatic patterns. For instance, despite similar perfusion levels, the brain and kidney are colonized at markedly different rates, indicating additional layers of molecular, biomechanical, and immunological specificity (Carrolo et al., 2024; Agrawal et al., 2025).

Recent advances have redefined the metastatic landscape to include a preparatory phase involving the formation of pre-metastatic niches (PMNs). Tumor-secreted factors, particularly extracellular vesicles (EVs), cytokines, and matrix-modifying enzymes, are capable of remodeling distant microenvironments before the arrival of metastatic cells, establishing fertile “soils” for subsequent colonization (Hu et al., 2023; Wang Y. et al., 2024; Peinado et al., 2017). These PMNs exhibit organ-specific molecular signatures, including fibronectin deposition, VEGFR1^+^ bone marrow-derived cell recruitment, and ECM stiffening, which collectively create permissive niches that guide organ-specific metastasis (Wang Y. et al., 2024; Gao et al., 2019).

Moreover, organotropism is profoundly influenced by immune contexture. Distinct immune niches across tissues such as the liver, lung, brain, and bone modulate whether disseminated tumor cells (DTCs) survive, enter dormancy, or proliferate (Wang Y. et al., 2024; Peinado et al., 2017; Deasy and Erez, 2022). Immune evasion is frequently mediated by EV-associated PD-L1, myeloid-derived suppressor cells (MDSCs), and tumor-associated macrophages (TAMs), establishing immunosuppressive milieus that facilitate metastatic outgrowth (Hu et al., 2023; Gao et al., 2019).

Additionally, the mechanical microenvironment, including extracellular matrix composition, tissue stiffness, and interstitial fluid pressure, exerts selective pressure on DTCs. Mechanotransduction pathways activated through integrins, focal adhesion kinases, and Rho GTPases enable tumor cells to adapt to the physical constraints of distant tissues, favoring survival and colonization in mechanically compatible organs (Fares et al., 2020; Deasy and Erez, 2022; Agrawal et al., 2025).

**FIGURE 1:**
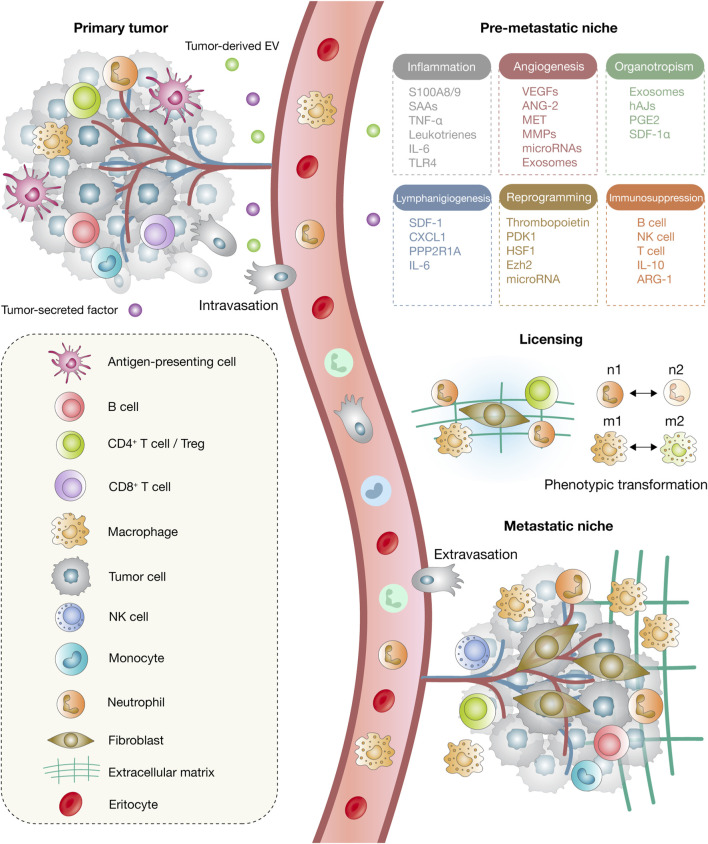
Model of metastatic progression. The process begins at the primary tumor site, which releases tumor-derived extracellular vesicles (EVs) and soluble factors that exert systemic effects on distant tissues. Circulating tumor cells (CTCs) subsequently disseminate through intravasation into the bloodstream and migrate to secondary organs. Within the pre-metastatic niche, a range of processes, including inflammation, angiogenesis, lymphangiogenesis, organotropism, immunosuppression, and stromal remodeling, are activated to establish a permissive microenvironment. During the licensing phase, phenotypic transitions occur, including the polarization of neutrophils from N1 to N2 and macrophages from M1 to M2 states, which facilitates extracellular matrix (ECM) remodeling and niche structuring. Finally, extravasation enables cancer cells to exit the circulation and colonize distant organs, where cancer-associated fibroblasts (CAFs) emerge as key components of the metastatic niche.

The emerging picture of metastatic organotropism is one of dynamic, reciprocal communication between disseminated tumor cells and organ-specific niches. This interaction is shaped by anatomical routes, vascular permeability, immune conditioning, stromal remodeling, and metabolic compatibility, all of which coordinate to dictate metastatic destiny (Gao et al., 2019; Dai et al., 2025). Understanding these determinants is not only critical for decoding the biology of metastasis but also essential for identifying therapeutic windows to intercept the metastatic cascade at organ-specific junctures. This multistep process is summarized in Figure 1, which illustrates the sequential stages of metastatic progression and the cellular and microenvironmental components involved.

## Anatomical determinants of metastatic organotropism

The dissemination of tumor cells to specific organs is profoundly influenced by anatomical factors that define the physical routes of metastatic spread. One of the most foundational principles is that circulating tumor cells (CTCs) often arrest in the first capillary bed downstream of the primary tumor. This anatomical filtration explains frequent patterns such as colorectal cancer metastasizing to the liver via the portal vein, and breast cancer spreading to the lungs via pulmonary circulation (Freitas et al., 2018; Obenauf and Massagué, 2015). However, vascular anatomy alone cannot fully explain organotropism, as organs with similar blood flow rates, such as the liver, kidney, and brain, display markedly different metastatic propensities (Obenauf and Massagué, 2015; Li et al., 2025).

The structure and dynamics of the vascular system impose mechanical constraints on tumor cells during intravascular transit. Capillary size, shear stress, and blood flow velocity critically determine whether a CTC will successfully arrest and extravasate. For instance, regions of low shear flow promote CTC adhesion and transmigration, while high-flow vessels inhibit stable attachment (Sturgess et al., 2023; Wirtz et al., 2011). Follain et al. demonstrated that vascular flow dynamics are not passive highways but active determinants of extravasation success, as shown in zebrafish and murine models (Freitas et al., 2018).

Additionally, the architecture and rigidity of capillaries present a mechanical barrier that CTCs must overcome. As tumor cells enter circulation, they face size restrictions and must deform substantially to pass through endothelial junctions in narrow capillaries, especially in the brain, bone, and lungs (Sturgess et al., 2023; Katt et al., 2018). This selects for cells with high deformability or the capacity to remodel the endothelium through proteolytic enzymes and physical force (Wirtz et al., 2011; Li et al., 2025).

The lymphatic system offers an alternative anatomical pathway, characterized by low pressure and less stringent structural barriers. Many tumors, especially those of epithelial origin such as breast and melanoma, metastasize via the lymphatic network, where CTCs experience reduced shear stress and immune surveillance compared to the bloodstream (Sturgess et al., 2023; Katt et al., 2018; Liu et al., 2024).

Anatomical proximity also contributes to site-specific colonization. The concept of “Batson’s venous plexus” has been invoked to explain how prostate cancer spreads to the axial skeleton by circumventing pulmonary filtration, enabled by retrograde venous flow in the valveless vertebral venous system (Obenauf and Massagué, 2015; Li et al., 2025). This phenomenon highlights that not all metastases follow a strictly linear or forward-flowing vascular path.

Tissue-specific stromal architecture further determines colonization efficiency. Dense extracellular matrix (ECM) components such as collagen, laminin, and fibronectin create mechanical and biochemical barriers in certain organs, while inflamed or pre-conditioned microenvironments are more permissive. Tumor-associated fibroblasts contribute to matrix remodeling and facilitate local invasion, particularly in mammary tumors where stromal stiffness enhances tumor cell motility and intravasation (Wirtz et al., 2011; Egeblad et al., 2010; Li et al., 2025).

Moreover, organ-specific vascular permeability affects metastatic access. Tumors may preferentially colonize organs with fenestrated or leaky capillaries, such as the liver or bone marrow, where endothelial junctions are looser and easier to breach (Liu et al., 2024; Martínez-Jiménez et al., 2023). This vascular leakiness often precedes metastasis and is associated with pre-metastatic niche formation, influenced by primary tumor-secreted factors (Freitas et al., 2018; Zhan et al., 2023).

Finally, the mechanical microenvironment, including interstitial fluid pressure and tissue stiffness, guides the intravasation, transport, and colonization of metastatic cells. The “seed and soil” hypothesis, originally conceptualized to describe compatibility between tumor cells and organ microenvironments, must be interpreted within the constraints and biases imposed by anatomical and biomechanical landscapes (Obenauf and Massagué, 2015; Zhan et al., 2023; Martínez-Jiménez et al., 2023).

## Molecular mechanisms underlying organotropism in metastasis

Organotropism, the non-random distribution of metastases to specific distant organs, is a hallmark of advanced malignancy and reflects a complex interplay between tumor-intrinsic programs and microenvironmental cues of target tissues. Originally proposed by Stephen Paget in 1889 as the “seed and soil” hypothesis, this concept has been robustly validated by contemporary molecular research. It highlights that organ-specific metastasis is governed not only by the intrinsic characteristics of tumor cells (“seed”) but also by the supportive and receptive nature of the target organ microenvironment (“soil”) (Liu et al., 2024; Wang and Luo, 2021; Dai et al., 2025).

Metastatic cells exhibit high phenotypic plasticity, largely mediated by epithelial-to-mesenchymal transition (EMT), a process that enables tumor cells to migrate, invade, resist apoptosis, and evade immune surveillance. EMT is orchestrated by key transcription factors such as SNAIL, SLUG, ZEB1, and TWIST, which repress epithelial markers and activate mesenchymal gene expression. Upon reaching the secondary organ, mesenchymal-to-epithelial transition (MET) may facilitate colonization, indicating that phenotypic switching is essential for successful metastatic progression (Gao et al., 2019; Fares et al., 2020; Nussinov et al., 2025). Tumor cells also express homing receptors and adhesion molecules that determine tissue-specific tropism, for instance, CXCR4 promotes bone metastasis via interaction with CXCL12 in the bone marrow, while integrins like αvβ3 and α6β4 contribute to brain and lung colonization, respectively (Massagué et al., 2017; Zhan et al., 2023).

During dissemination, circulating tumor cells (CTCs) serve as key intermediates in organotropic spread. These cells exhibit extensive molecular heterogeneity and often display hybrid EMT states and stem-like properties. Their expression of surface markers such as EpCAM, CD44, and various integrins allows vascular arrest and extravasation into favorable microenvironments (Li et al., 2025). Importantly, the route of dissemination also shapes tropism; lymphatic spread, which is predominant in cancers like breast carcinoma and melanoma, involves VEGF-C, LYVE-1, and PROX1 to induce lymphangiogenesis and lymph node colonization, serving as a springboard for subsequent systemic dissemination (Leong et al., 2022; Zhan et al., 2023).

At the distant site, tumor cell survival and expansion depend on their capacity to adapt to the target organ’s unique microenvironment. Pre-metastatic niche formation, driven by tumor-derived extracellular vesicles such as exosomes enriched in integrins and immunomodulatory molecules like PD-L1, primes specific organs by promoting inflammation, immune suppression, vascular leakiness, and extracellular matrix remodeling. These conditions foster an environment conducive to colonization by incoming tumor cells (Liu et al., 2024; Nussinov et al., 2025; Massagué et al., 2017; Kyriakidis et al., 2025; Araujo-Abad et al., 2024; López-Cortés et al., 2021). Additionally, local stromal elements, such as osteoblasts in bone or astrocytes in the brain, actively contribute to metastatic outgrowth by providing supportive signals and promoting phenotypic adaptation (Gao et al., 2019).

Metabolic plasticity plays a crucial role in organ-specific colonization. Tumor cells reprogram their energy metabolism based on the nutrient and oxygen landscape of the target tissue (Li et al., 2025). For example, brain metastases rely more heavily on oxidative phosphorylation, whereas bone metastases adopt glycolytic profiles. The ability to resist oxidative stress is particularly critical, as it allows metastatic cells to withstand hostile microenvironments. Breast cancer subtypes with high cancer stem cell content and redox adaptability, such as triple-negative breast cancers (TNBCs), preferentially colonize the lung and brain, whereas luminal tumors tend to metastasize to the liver and bone (Wang and Luo, 2021; Dai et al., 2025). In the lung, metastases occur in up to 54% of patients with advanced disease, a frequency facilitated by the organ’s physical and immune properties (Xiao et al., 2025). Recent work has revealed that pulmonary aspartate acts as an extracellular signaling molecule that activates the N-methyl-D-aspartate receptor in disseminated breast cancer cells, triggering a CREB-dependent program that upregulates deoxyhypusine hydroxylase and promotes eIF5A hypusination. This translational program, with TGF-β signaling as a central hub, enhances collagen synthesis and drives aggressiveness of lung metastases (Doglioni et al., 2025).

Finally, organotropism reflects clonal selection and epigenetic reprogramming. Single-cell transcriptomic and spatial profiling studies have shown that distinct subclones within a primary tumor exhibit variable capacities for organ-specific metastasis. These clones undergo selective pressure at secondary sites, leading to epigenetic remodeling, such as chromatin alterations and DNA methylation changes, that activate gene expression programs tailored to the target organ (Fares et al., 2020; Nussinov et al., 2025; Massagué et al., 2017). Recent cytogenetic analyses in colorectal cancer demonstrated that organ-biased copy number alterations are acquired early in tumor evolution, with recurrent events enriched in liver and brain metastases, highlighting the genomic basis of organotropic potential (Golas et al., 2025). Recent technological advances have expanded these insights: the MetMap barcoding atlas of 500 human cancer cell lines revealed reproducible organ-specific metastatic patterns linked to PI3K signaling and lipid metabolism in brain colonization (Jin et al., 2020); single-cell RNA-seq has identified translational rewiring programs in lung-tropic clones, indicating that metastatic fitness relies on both transcriptional and post-transcriptional control (Laughney et al., 2020); and high-resolution proteomics and metabolomics have exposed tissue-specific metabolic dependencies that determine survival under oxidative stress and nutrient scarcity (Wang H. et al., 2024; Gundogan et al., 2024). Together, these platforms demonstrate that organotropism is not only anatomically and immunologically determined but also a consequence of molecular programs uncovered by single-cell and multi-omic technologies.

In summary, organotropism in metastatic cancer is not governed by a single determinant but rather emerges from the coordinated interaction of tumor-intrinsic properties, immune evasion, metabolic reprogramming, and mechanistic insights provided by advanced technologies such as single-cell sequencing, spatial transcriptomics, proteomics, and metabolomics, ultimately dictating the spatial distribution of metastases across the body.

## Tumor-derived extracellular vesicles and their role in pre-metastatic niche formation

Tumor-derived EVs, including exosomes and microvesicles, have emerged as central regulators of pre-metastatic niche (PMN) formation. These nano-sized vesicles, secreted by primary tumors into the circulation, carry a diverse cargo of proteins, lipids, nucleic acids, and metabolites that reflect the molecular signature of their tumor of origin (Wang Y. et al., 2024; Kumar et al., 2024). Once they reach distant organs, EVs reprogram the local microenvironment to become permissive to future metastatic colonization (Yang et al., 2021; Rezaie et al., 2022). Mechanistically, tumor-derived exosomes whose cargo is tuned by matrix stiffness reprogram distant glucose metabolism to create a glucose-enriched lung pre-metastatic niche before cancer cell arrival (Zhao et al., 2025).

One key mechanism through which EVs shape PMNs is via their integrin expression profiles. Exosomes derived from highly metastatic tumors display specific integrins that mediate selective organotropism. For instance, exosomal integrin α6β4 and α6β1 promote lung tropism, while αvβ5 directs vesicle uptake by Kupffer cells in the liver. Upon uptake, these resident cells secrete pro-inflammatory cytokines, extracellular matrix remodeling enzymes, and angiogenic factors, effectively pre-conditioning the organ for incoming tumor cells (Rezaie et al., 2022; Zhao et al., 2021). This integrin-mediated targeting of EVs has been functionally validated in multiple models of breast, pancreatic, and melanoma metastasis (Zhang et al., 2024).

Tumor-derived EVs also mediate profound immunomodulatory effects. By delivering immune-suppressive molecules such as TGF-β, IL-10, or PD-L1, they induce polarization of macrophages toward a tumor-associated (M2-like) phenotype, expansion of myeloid-derived suppressor cells (MDSCs), and suppression of cytotoxic T cell and NK cell responses (Clancy and D’Souza-Schorey, 2023; Sun et al., 2018). These immune alterations help generate a tolerogenic microenvironment that not only supports metastatic cell survival but also facilitates immune escape during the early phases of colonization (Zhao et al., 2021).

In the vasculature, EVs promote vascular leakiness and angiogenesis, creating physical and biochemical conditions favorable for DTC extravasation. This occurs through the upregulation of matrix metalloproteinases (MMP2/9), vascular endothelial growth factor (VEGF), and inflammatory cytokines in endothelial cells upon EV uptake. Notably, increased permeability and neovascularization are hallmarks of EV-induced niche remodeling in organs such as the lung, liver, and brain, where fenestrated or sinusoidal capillaries offer fertile ground for metastasis (Ghoroghi et al., 2021; Rezaie et al., 2022).

The metabolic landscape of the target organ is also modified by tumor EVs. Certain vesicles carry metabolites, mitochondrial components, or miRNAs that regulate redox balance and glucose metabolism in stromal or endothelial cells, thereby facilitating colonization by metabolic reprogramming (Zhang et al., 2024; Yang et al., 2021). For example, EVs from breast cancer cells have been shown to enhance oxidative stress tolerance in recipient lung fibroblasts, creating a niche more hospitable to subsequent tumor cell arrival (Clancy and D’Souza-Schorey, 2023).

Moreover, EVs play a role in awakening dormant niches. Dormant DTCs in bone marrow, liver, or brain may remain quiescent for years, and signals from EVs can disrupt this state. Specifically, vesicles bearing pro-inflammatory miRNAs or ECM-remodeling enzymes like LOX or TIMP3 alter stromal signaling and can trigger the reactivation of previously dormant cells (Tan et al., 2021; Kumar et al., 2024; Ghoroghi et al., 2021).

From a translational perspective, tumor-derived EVs are promising biomarkers for early detection of metastasis and for monitoring disease progression. Their stability in circulation and the tissue specificity of their cargo enable non-invasive profiling through liquid biopsy. EVs can be isolated from plasma, urine, or cerebrospinal fluid, and their proteomic or miRNA content often correlates with metastatic risk, site preference, and therapeutic resistance (Wang Y. et al., 2024; Clancy and D’Souza-Schorey, 2023; Sun et al., 2018).

In summary, tumor-derived extracellular vesicles are not passive byproducts of tumor biology but active architects of metastatic progression. Through organ-targeted delivery of signaling molecules, metabolic regulators, and immunomodulatory factors, EVs orchestrate the formation of pre-metastatic niches. Understanding their role in niche education and organotropism offers both mechanistic insight and novel therapeutic targets in the prevention and management of metastatic cancer.

## Organ-specific immune niches in metastatic progression

The formation and outgrowth of metastases are profoundly influenced by the immune microenvironments of distant organs, which vary in their cellular composition, immunological tone, and responsiveness to tumor-derived signals. These organ-specific immune niches play a central role in determining whether disseminated tumor cells (DTCs) are eliminated, enter dormancy, or progress to overt metastases (Janssen et al., 2017; Grant and Ferrer, 2025; Liu et al., 2024).

A key step in this process is the establishment of pre-metastatic niches (PMNs), which are primed by factors secreted from the primary tumor even before the arrival of CTCs. These factors include EVs, cytokines, and exosomes that modulate the immune landscape of future metastatic sites by enhancing vascular permeability, suppressing local immunity, and promoting the recruitment of immunosuppressive cells such as regulatory T cells (Tregs), myeloid-derived suppressor cells (MDSCs), and tumor-associated macrophages (TAMs) (Correia, 2023; Salmon et al., 2019). In organs such as the lung, liver, and bone, this immunomodulation facilitates CTC extravasation and supports initial survival (Grant and Ferrer, 2025; Liu et al., 2024). Recent *in vivo* studies revealed that transient activation of the aryl hydrocarbon receptor (AHR) in lung macrophages precedes metastatic seeding and induces PD-L1–dependent Treg expansion, establishing an immunosuppressive pre-metastatic niche (Jiang et al., 2024).

Each organ possesses a unique immune architecture that influences metastatic progression. The lung, for example, is enriched in alveolar macrophages and neutrophils, which can paradoxically promote metastasis by forming neutrophil extracellular traps (NETs) or shielding tumor cells from cytotoxic T cells (Grant and Ferrer, 2025; Wang Y. et al., 2024). In the liver, Kupffer cells and hepatic stellate cells establish a tolerogenic immune environment that impairs anti-tumor T cell responses, allowing metastatic seeding and outgrowth (Salmon et al., 2019; Patras et al., 2023). In contrast, the bone marrow niche is rich in immunosuppressive mesenchymal stromal cells and resident macrophages that support either dormancy or activation of DTCs depending on local cues (El-Kenawi et al., 2020).

Immune-mediated dormancy represents a pivotal checkpoint in metastatic progression. Following extravasation, many DTCs enter a prolonged quiescent state, maintained by immune surveillance involving CD8^+^ T cells, NK cells, and the absence of pro-inflammatory signaling (Janssen et al., 2017; El-Kenawi et al., 2020). However, systemic inflammation, aging, or therapy-induced immunosuppression can disrupt this equilibrium, triggering DTC reactivation and overt metastasis. Mathematical and experimental models demonstrate that micrometastatic populations can persist for years under immune constraint before escaping due to stochastic fluctuations in immune pressure (Grant and Ferrer, 2025; Shrestha et al., 2025).

Importantly, the immune composition of metastatic sites is highly dynamic and plastic. Chemotherapy, radiotherapy, and surgical stress can inadvertently alter immune cell recruitment and function within metastatic niches, either enhancing immune clearance or promoting immune evasion (Janssen et al., 2017; Grant and Ferrer, 2025; Wang Y. et al., 2024). For instance, surgery-induced inflammation in lung and liver increases neutrophil recruitment and immunosuppressive cytokine release, creating a favorable environment for DTC reactivation (Patras et al., 2023; Grant and Ferrer, 2025). Additionally, the interplay between tumor cells and immune cells is bidirectional. Tumor-derived exosomes carry PD-L1, inflammatory mediators, and immunosuppressive microRNAs that actively suppress T cell function in future metastatic sites (Correia, 2023; Salmon et al., 2019; Liu et al., 2024). Furthermore, exosomal integrins dictate the organotropism of these vesicles and the immune conditioning of specific tissues such as the brain, lungs, and liver (Patras et al., 2023).

In summary, organ-specific immune niches serve as critical modulators of metastatic progression by orchestrating a balance between immune elimination, dormancy, and escape. These niches are dynamically shaped by tumor-derived signals, host immunity, and therapeutic interventions, representing both a challenge and an opportunity for intervention. Targeting immunosuppressive components within these niches may offer novel strategies to prevent metastasis or maintain DTCs in a dormant, non-proliferative state (Janssen et al., 2017; Shrestha et al., 2025).

## Organ-specific microenvironments and their role in selective colonization

The colonization of distant organs by disseminated tumor cells (DTCs) is not merely a function of anatomical accessibility or passive entrapment in capillary beds. Rather, it is a selective and dynamic process governed by the unique molecular, cellular, and structural features of each organ microenvironment. The interaction between metastatic “seeds” and organ-specific “soils” ultimately determines whether disseminated cells persist, enter dormancy, or initiate macrometastatic outgrowth (Wang and Luo, 2021; Nguyen et al., 2009).

A first determinant is the vascular anatomy and hemodynamic flow of the target organs. Organs such as the liver, lung, and bone marrow, frequent sites of metastasis, possess fenestrated or sinusoidal capillaries that allow easier extravasation of tumor cells. However, vascular exposure alone does not guarantee successful colonization. The kidney and brain receive comparable blood flow but are less commonly colonized due to restrictive stromal and immune features (Schild et al., 2018; Smith and Kang, 2017; Obenauf and Massagué, 2015). These differences underscore the need for tumor cells to adapt to local environmental constraints beyond physical accessibility.

Once DTCs extravasate into tissue parenchyma, they must contend with hostile environments characterized by oxidative stress, immune surveillance, and nutrient scarcity. The majority of DTCs undergo apoptosis or are eliminated by resident immune cells. Colonization is thus considered the rate-limiting step in metastasis (Massagué and Obenauf, 2016). Only rare cells with context-specific adaptations survive and proliferate. Organ-specific stromal cells, including fibroblasts, endothelial cells, and perivascular niches, can either suppress or promote metastatic outgrowth depending on the microenvironmental context (Pranzini et al., 2025; Nguyen et al., 2009). Recent evidence shows that primary breast tumors actively remodel the extracellular matrix of the lung before metastasis, increasing fibrillar collagen deposition, basement membrane disruption, and vascular leakiness, thereby priming the pulmonary niche for colonization (Cai et al., 2023).

In the bone marrow, for example, mesenchymal stem cells and osteoblasts secrete CXCL12, TGF-β, and IL-6, which support the dormancy and eventual outgrowth of DTCs through integrin signaling and metabolic adaptation (Wang and Luo, 2021). In the liver, hepatic stellate cells and Kupffer cells release pro-inflammatory cytokines and ECM remodeling enzymes that can facilitate colonization by promoting epithelial plasticity and immune evasion (Smith and Kang, 2017). In contrast, the lung exhibits a dense network of alveolar macrophages and high oxygen tension that often impairs colonization unless tumor cells suppress immune activation or co-opt resident stromal signals (Schild et al., 2018; Pranzini et al., 2025).

Dormancy is a key feature in organs like the bone, brain, and liver, where DTCs may remain quiescent for years before reactivation. Factors such as BMP-7, thrombospondin-1, and GAS6/TAM signaling maintain DTCs in a non-proliferative state. This is not simply a passive process; rather, the microenvironment actively imposes quiescence, and changes in local signals can trigger reactivation (De Visser and Joyce, 2023; Maeshiro et al., 2021). For example, a shift in the immune or inflammatory milieu, such as neutrophil influx or ECM stiffening, can awaken dormant cells and promote micrometastatic outgrowth (Pranzini et al., 2025; Massagué and Obenauf, 2016).

Additionally, the physical characteristics of the metastatic niche, including the composition and stiffness of the extracellular matrix (ECM), can significantly affect the efficiency of tumor cell colonization. In the brain, for example, low ECM density and the presence of astrocytic endfeet create a metabolically restrictive niche that only select cancer cell clones can adapt to. Conversely, bone is rich in calcium and growth factors released during osteoclastic bone resorption, which can stimulate cancer proliferation (De Visser and Joyce, 2023; Wang and Luo, 2021; Smith and Kang, 2017).

The temporal dynamics of colonization also vary. Breast cancer metastases to the brain and bone often remain clinically silent for years, while metastases from lung cancer typically emerge rapidly, indicating that colonization is governed by both tumor-intrinsic programs and the permissiveness or latency-inducing properties of the microenvironment (Nguyen et al., 2009; Obenauf and Massagué, 2015). CTC clusters have also been shown to display enhanced metastatic seeding, particularly in organs like the brain and lung, due to their increased resistance to shear stress and immune attack (Maeshiro et al., 2021).

In summary, organ-specific colonization reflects an active crosstalk between DTCs and their new microenvironment. It involves immune modulation, mechanical adaptation, metabolic reprogramming, stromal co-option, and, in some cases, long-term dormancy. Understanding how distinct tissues either suppress or support colonization offers a powerful framework for developing site-specific anti-metastatic therapies.

## Organotropism patterns across major cancer types and clinical implications

**FIGURE 2:**
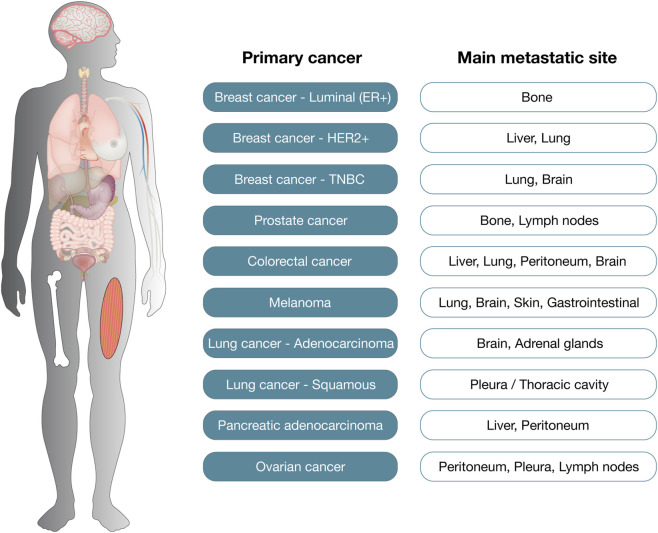
Preferred metastatic sites across major cancer types.

Organotropism, the propensity of metastatic tumor cells to colonize specific distant organs, is a consistent and biologically meaningful feature observed across diverse cancer types. These patterns are not random but reflect the intricate interaction between tumor cell-intrinsic programs and the permissiveness of organ-specific microenvironments (Jee et al., 2024; Huppert et al., 2022). Understanding these preferences has enabled the development of important tools for clinical surveillance, prognostication, and targeted therapy (Ganguly and Kimmelman, 2023). Importantly, Figure 2 summarizes the preferred metastatic sites across major cancer types.

Breast cancer is among the most well-characterized in terms of organotropic behavior. Subtypes defined by molecular profiles, luminal A, luminal B, HER2-enriched, and TNBC, display distinct metastatic trajectories (López-Cortés et al., 2020b; López-Cortés et al., 2018). Luminal subtypes, which express estrogen receptors, preferentially metastasize to bone. This is thought to be driven by chemokine receptor CXCR4 expression, which mediates homing to CXCL12-rich bone marrow niches, and by interactions with osteoblasts and bone stromal cells that create a supportive niche (Huppert et al., 2022; Skaro et al., 2021). HER2-enriched tumors tend to colonize visceral organs such as the liver and lung, whereas TNBC, which lacks hormone receptor expression, is more prone to metastasize to the lung and brain. This tropism is associated with a mesenchymal phenotype, enhanced oxidative stress tolerance, and upregulation of matrix-degrading enzymes like MMP1 and cathepsins (Shi et al., 2025; Obenauf and Massagué, 2015).

Prostate cancer displays a striking preference for bone metastases, frequently forming osteoblastic rather than osteolytic lesions. This specificity is driven in part by tumor-secreted endothelin-1, bone morphogenetic proteins (BMPs), and androgen receptor (AR) signaling that promotes a bone-forming environment (Paz-Y-Miño et al., 2016). The bone tropism of prostate cancer also involves integrin-mediated interactions and secretion of RANKL, which activates osteoblasts and supports metastatic outgrowth (Skaro et al., 2021; Gupta and Massagué, 2006).

Colorectal cancer (CRC) typically follows a sequential metastatic route dictated by its anatomical drainage through the portal vein. The liver is the primary metastatic site, followed by the lungs and, less frequently, the brain. However, liver tropism is not only anatomical; the liver’s immunotolerant microenvironment and its population of Kupffer cells and hepatic stellate cells are exploited by tumor cells to evade immune destruction and establish metastases (Ganguly and Kimmelman, 2023; Gupta and Massagué, 2006; López-Cortés et al., 2020c). Certain mutations, such as in KRAS or BRAF, also influence the likelihood and aggressiveness of liver metastases (Huppert et al., 2022).

Melanoma exhibits one of the most versatile metastatic spectra among solid tumors, colonizing the lung, brain, skin, gastrointestinal tract, and distant lymph nodes. This broad organotropism reflects melanoma’s origin from neural crest cells, its high degree of phenotypic plasticity, and the expression of a diverse array of chemokine receptors (e.g., CCR7, CXCR4) and integrins (Ganguly and Kimmelman, 2023; Obenauf and Massagué, 2015). Notably, brain metastasis in melanoma correlates with elevated expression of PTEN loss and BRAF mutations, and occurs more frequently in patients with high circulating tumor cell counts and elevated exosomal PD-L1 (Gu et al., 2024).

Lung cancer, particularly non-small cell lung cancer (NSCLC), demonstrates subtype-specific metastatic preferences. Lung adenocarcinomas more commonly metastasize to the brain and adrenal glands, while squamous cell carcinomas tend to remain within the thoracic cavity. EGFR-mutant NSCLCs frequently metastasize to the brain, facilitated by disruption of the blood–brain barrier and exosome-mediated pre-metastatic niche formation (Shi et al., 2025; Obenauf and Massagué, 2015).

Pancreatic ductal adenocarcinoma (PDAC) is another cancer with aggressive organotropism, predominantly metastasizing to the liver and peritoneum. The liver’s sinusoidal structure and abundant macrophage populations contribute to the formation of a pre-metastatic niche through recruitment of bone marrow-derived cells and uptake of tumor-derived exosomes (Obenauf and Massagué, 2015; Ganguly and Kimmelman, 2023). Similarly, ovarian cancer primarily disseminates within the peritoneal cavity and colonizes the omentum, driven by mesothelial cell–tumor interactions and inflammatory cytokine gradients (Coulton and Turajlic, 2022).

From a clinical standpoint, recognition of these patterns informs surveillance and treatment strategies. For example, patients with HER2+ breast cancer benefit from liver imaging, while those with prostate cancer require bone scans. Brain imaging is essential in melanoma, lung cancer, and TNBC due to their neurotropic tendencies. Furthermore, therapies tailored to metastatic niches, like bisphosphonates and denosumab for bone metastases, or immune checkpoint inhibitors in brain metastases, demonstrate how organotropism can be therapeutically exploited (Huppert et al., 2022; Ganguly and Kimmelman, 2023; Gupta and Massagué, 2006).

Importantly, multi-omic and machine learning approaches are now being used to predict metastatic trajectories (López-Cortés et al., 2020a). An analysis integrating transcriptomic data from TCGA classified primary tumors according to their predicted metastatic destinations, revealing shared gene expression patterns among tumors that metastasize to the same organ, regardless of their tissue of origin (Skaro et al., 2021). Similarly, Jiang et al. developed the MetaNet computational framework that integrates clinical and genomic features to stratify patients by risk and organ-specific metastasis likelihood, enabling personalized surveillance regimens and early therapeutic interventions (Jiang et al., 2021).

In conclusion, organotropism is a reproducible and clinically actionable phenomenon across cancer types. Mapping these patterns not only aids in anticipating metastatic spread but also uncovers shared molecular mechanisms that can be targeted to disrupt colonization. As our ability to model and predict organ-specific dissemination improves, organotropism will become a critical axis in precision oncology.

## Therapeutic opportunities and challenges in targeting organotropism in metastasis

Targeting organotropism, the preference of metastatic tumor cells for specific organs, offers a novel and potentially transformative axis for therapeutic intervention. As the majority of cancer deaths result from metastatic disease rather than primary tumors, disrupting the organ-specific dissemination of tumor cells could significantly improve long-term survival. Nonetheless, the biological complexity of metastatic progression and structural barriers within clinical research frameworks pose significant obstacles to translating this strategy into effective treatments (Pang and Wang, 2024; Li et al., 2025; Liu et al., 2024).

The metastatic process is not merely a stochastic event but is orchestrated by molecular determinants that enable tumor cells to home to, survive in, and colonize specific organ microenvironments. These interactions unfold sequentially, starting with tumor cell detachment, followed by intravasation, survival in the bloodstream, adhesion to the target endothelium, extravasation, and ultimately, adaptation to the new microenvironment. Each step offers potential therapeutic targets, but the organ-specificity of many interactions demands tailored strategies that account for unique vascular, immune, and stromal features in each tissue (Dai et al., 2025; Anderson et al., 2019; Fidler and Kripke, 2015).

One critical barrier to intervention is that most metastases have already been initiated prior to clinical detection, with micrometastatic disease often seeded at early stages. Thus, preventing the progression of these dormant niches into overt metastases represents a major opportunity. The pre-metastatic niche (PMN), a primed microenvironment formed in target organs before tumor cell arrival, is a focal point of this strategy. Tumor-derived extracellular vesicles (EVs), cytokines, and remodeling enzymes initiate PMN formation by altering vascular permeability, recruiting bone marrow-derived suppressive cells, and reprogramming stromal and immune elements. Therapeutic efforts aimed at disrupting these early changes, such as blocking EV uptake, inhibiting ECM crosslinking via lysyl oxidase (LOX), or neutralizing niche-activating factors like S100A8/9, have demonstrated efficacy in preclinical models (Pang and Wang, 2024; Li et al., 2025; Zhou et al., 2020).

Despite this promise, the temporal complexity and tissue-specific variation in PMN formation pose serious challenges. The molecular triggers that govern PMN development are highly dynamic and differ between organs. For example, liver pre-metastatic niches are rich in fibronectin, VEGFR1+ bone marrow-derived cells, and Kupffer cell-derived inflammatory cues, while lung PMNs often involve neutrophil recruitment and pro-inflammatory signaling cascades. This heterogeneity complicates the design of generalized therapies and necessitates biomarkers capable of identifying PMN activity in specific organs (Shi et al., 2024; Zhou et al., 2020).

Another intervention point is the blockade of tumor cell adhesion to the target endothelium. Adhesion molecules such as integrins, selectins, and CD44 mediate organotropic interactions with vascular beds and perivascular niches. Recent advances have shown that the pharmacological inhibition of these adhesion processes can reduce organ-specific colonization. Quantitative metrics such as the adhesion/inhibition ratio (AIR) provide a functional readout of compound efficacy in preventing tumor–endothelial engagement under shear flow conditions. These approaches have demonstrated selectivity in disrupting hepatic versus pulmonary metastasis, depending on the molecular target (Parker et al., 2022; Xie et al., 2022).

However, translating anti-organotropism strategies into clinical practice requires a fundamental reconfiguration of drug development paradigms. Traditional preclinical models and clinical trials are biased toward detecting tumor shrinkage, often failing to evaluate changes in metastatic potential. Agents that may halt colonization or suppress metastatic outgrowth without reducing primary tumor size are frequently dismissed due to perceived inefficacy by RECIST or similar criteria (Pang and Wang, 2024; Xie et al., 2022; Anderson et al., 2019). Furthermore, few clinical trials stratify patients based on organ-specific metastasis risk, thereby diluting signals of efficacy in subpopulations who might benefit most from targeted intervention.

Another critical challenge lies in the biological divergence between primary tumors and their metastases. Metastatic clones often exhibit distinct transcriptional and metabolic profiles shaped by their interaction with the host organ microenvironment. These differences result in therapeutic resistance that cannot be predicted based on the characteristics of the primary tumor alone. For example, stromal signals in bone metastases induce osteomimicry in prostate and breast cancer cells, while brain metastases exhibit metabolic adaptations favoring oxidative phosphorylation and immune evasion. Therapeutically targeting these adaptations, by disrupting niche-specific integrin signaling or modulating stromal-derived growth factors, holds potential for enhanced efficacy, but it necessitates a detailed, organ-specific understanding of the underlying mechanisms (Liu et al., 2024; Parker et al., 2022; Fidler and Kripke, 2015). In support of this, an *in vivo* CRISPRa screen recently identified acyl-CoA binding protein (ACBP) as a metabolic driver of bone metastasis, coupling lipid metabolism to metastatic fitness and nominating a targetable vulnerability for organ-specific intervention (Teng et al., 2025).

The absence of real-time biomarkers to monitor PMN activity and metastatic colonization further complicates clinical translation. Liquid biopsy tools, such as assays for circulating EVs, tumor-derived DNA, and niche-associated proteins (e.g., periostin, fibronectin), offer promise for detecting metastatic risk before radiographic evidence emerges. In particular, exosomal integrins and pre-metastatic niche signatures in plasma have shown potential in stratifying patients likely to develop lung or liver metastases (Li et al., 2025; Dai et al., 2025; Shi et al., 2024).

Targeting organotropism also raises safety and specificity concerns (Zhou et al., 2020). Because many of the molecules involved in PMN formation or endothelial adhesion are also active in wound healing, immune homeostasis, or normal organ development, systemic inhibition may produce unintended side effects. Thus, local delivery methods or prodrug formulations that activate in the niche environment are under investigation to mitigate off-target effects (Shi et al., 2024; Fidler and Kripke, 2015).

Finally, coordinated action across research consortia, funding bodies, regulatory agencies, and pharmaceutical developers is essential to overcome inertia in the metastatic drug development space. A revised framework is needed to prioritize metastasis-specific endpoints, incorporate organ-specific risk stratification, and support longitudinal trials that assess not only overall survival but also time to metastatic progression and organ-specific relapse.

## Conclusion and future perspectives

Organotropism in metastatic cancer is a non-random and biologically regulated phenomenon governed by the interplay between tumor-intrinsic traits and the unique features of distant organ microenvironments. Evidence from recent studies confirms that the pre-metastatic niche is not a passive setting but is actively shaped by the primary tumor before metastatic seeding. Tumor-derived EVs, cytokines, and matrix-modifying enzymes precondition specific organs by modulating immune components, enhancing vascular permeability, and reprogramming stromal elements (Li et al., 2025; Nussinov et al., 2025; Kumar et al., 2024; Araujo-Abad et al., 2024).

Distinct immune architectures across organs define how disseminated tumor cells (DTCs) are received and whether they are eliminated, held in dormancy, or allowed to proliferate. For example, the liver’s tolerogenic environment and the lung’s neutrophil-enriched milieu provide immunological contexts that promote metastatic progression, while the brain enforces stringent entry and survival requirements due to its immune-privileged status and blood–brain barrier (Nussinov et al., 2025; Obenauf and Massagué, 2015; Shi et al., 2024). These findings underscore the necessity of tailoring therapeutic strategies to specific organ immune environments.

The phenotypic plasticity of metastatic cells, particularly their ability to undergo epithelial-to-mesenchymal and mesenchymal-to-epithelial transitions, allows them to adapt dynamically to different organ niches. Tumor heterogeneity also plays a key role, as only subclones with specific integrin profiles, metabolic flexibility, and immunoevasive features are capable of colonizing particular organs (Schild et al., 2018; Liu et al., 2024). Notably, brain metastases often arise from clones with enhanced oxidative phosphorylation capacity and PD-L1-enriched EVs that suppress local immune responses (Obenauf and Massagué, 2015; Liu et al., 2024).

Future directions should prioritize the development of predictive biomarkers for organ-specific metastasis using liquid biopsy tools such as circulating EVs and CTC profiling. These tools enable real-time monitoring of tumor evolution and metastatic risk (Gu et al., 2024; Obenauf and Massagué, 2015). Additionally, targeting the earliest steps of pre-metastatic niche formation, such as blocking integrin-mediated EV uptake or inhibiting ECM remodeling enzymes like LOX, holds promise for preventing metastatic outgrowth before clinical manifestation (Li et al., 2025; Kumar et al., 2024; Shi et al., 2024).

Equally important is the advancement of physiologically relevant *in vivo* models that mimic human metastatic dissemination patterns, including organ-specific immune niches and stromal contexts. Such models are essential to evaluate therapeutic efficacy beyond primary tumor shrinkage and to capture metastasis-suppressive effects often missed by traditional endpoints (Schild et al., 2018; Liu et al., 2024).

In clinical translation, integrating organotropism into patient management could redefine surveillance and treatment paradigms. Personalized risk stratification, based on tumor genotype, epigenetics, metastatic signatures, niche biomarkers, circadian rhythm dysruptions, and microbiome dysfunction may guide early interventions to delay or prevent metastasis (Pérez-Villa et al., 2023; Bautista et al., 2025b; Ocaña-Paredes et al., 2024; Bautista et al., 2025a). Ultimately, by deciphering the mechanisms of organ-specific colonization, metastasis may be transformed from an inevitable consequence to a tractable and preventable process (Zhang et al., 2024; Kumar et al., 2024; Obenauf and Massagué, 2015).
